# A Case Report of Cyanosis With Refractory Hypoxemia: Is It Methemoglobinemia?

**DOI:** 10.7759/cureus.32053

**Published:** 2022-11-30

**Authors:** Sangita D Kamath, Shashank Sunny, Ajatshatru Upadhyay

**Affiliations:** 1 Internal Medicine, Tata Main Hospital, Jamshedpur, IND; 2 General Medicine, Tata Main Hospital, Jamshedpur, IND

**Keywords:** methylene blue, hypoxia, methemoglobinemia, cyanosis, dapsone

## Abstract

Dapsone is used in the treatment of a variety of dermatological conditions and prophylaxis of opportunistic infections. However, if consumed at a dose of more than 200 mg/day, it can cause methemoglobinemia, a condition characterized by elevated methemoglobin levels in the blood; methemoglobin is an abnormal form of hemoglobin, containing iron in the ferric state (Fe^3^^+^) rather than the reduced ferrous form (Fe^2^^+^) found in hemoglobin. A small amount of it is produced in the body due to oxidant damage to the red blood cells. Methemoglobinemia can cause varied clinical manifestations involving the cardio-respiratory and nervous systems depending upon the level of methemoglobin. While it could be congenital, it is commonly caused by exposure to drugs that cause oxidation of hemoglobin, such as benzocaine, dapsone, and nitrates. We report a case of dapsone-induced methemoglobinemia in a previously healthy young female who had consumed 15 tablets of dapsone 100 mg with suicidal intent. She presented with central cyanosis, breathlessness, and altered sensorium after five days of consumption. While the pulse-oximeter showed oxygen saturation (SaO_2_) of 84%, arterial blood gas (ABG) analysis showed partial pressure of oxygen (PaO_2_) of 427 mmHg and SaO_2_ of 98%. This "saturation gap" occurred due to the presence of the abnormal hemoglobin variant. Her cyanosis did not improve despite giving 100% supplemental oxygen. There was no cardiac or respiratory cause to account for her cyanosis. Her methemoglobin level was 45.8%. She was successfully treated with specific antidote methylene blue, mechanical ventilation, and other symptomatic measures. The purpose of this presentation is to help clinicians recognize this condition early, because, if left untreated, it might prove fatal. The diagnostic clues include refractory hypoxemia, central cyanosis in the absence of cardiac and respiratory causes, saturation gap, and chocolate-colored blood.

## Introduction

Methemoglobinemia is a potentially life-threatening condition caused by the oxidation of the ferrous form of iron (Fe^2+^) in the heme component of hemoglobin to the ferric form (Fe^3+^). As ferric iron cannot bind to and transport oxygen, this results in the decreased oxygen-carrying capacity of hemoglobin, functional anemia, and thus tissue hypoxia [1]. Normally, it is present in a very small amount of less than 1% in the blood. It can result from congenital or acquired causes. The congenital causes include autosomal-dominant mutations in globin genes near the heme iron that produce hemoglobin M or autosomal-recessive mutations in the enzyme cytochrome b5 reductase (CYB5R). Acquired causes are more common and include exposure to drugs that cause direct oxidation of hemoglobin, such as benzocaine and prilocaine, those that cause indirect oxidation, such as nitrates, and those that require metabolic activation, such as dapsone and aniline [2]. Clinical manifestations depend on the level of methemoglobin. We discuss a case of methemoglobinemia caused by the consumption of a toxic dose of dapsone (1500 mg) with suicidal intent.

## Case presentation

A 16-year-old female was admitted for a bluish discoloration of fingertips, toes, and tongue along with breathlessness, palpitation, and altered sensorium of two days' duration. She had consumed 15 tablets of dapsone after a quarrel with her mother five days back. She had been taken to a local hospital after she complained of vomiting and abdominal pain. There she had been given a stomach wash and symptomatic treatment and discharged on the second day. On the fifth day after the incident, she was brought to our hospital with the above-mentioned symptoms. There was no history of fever or cough and no past history of any significant medical ailment.

**Figure 1:**
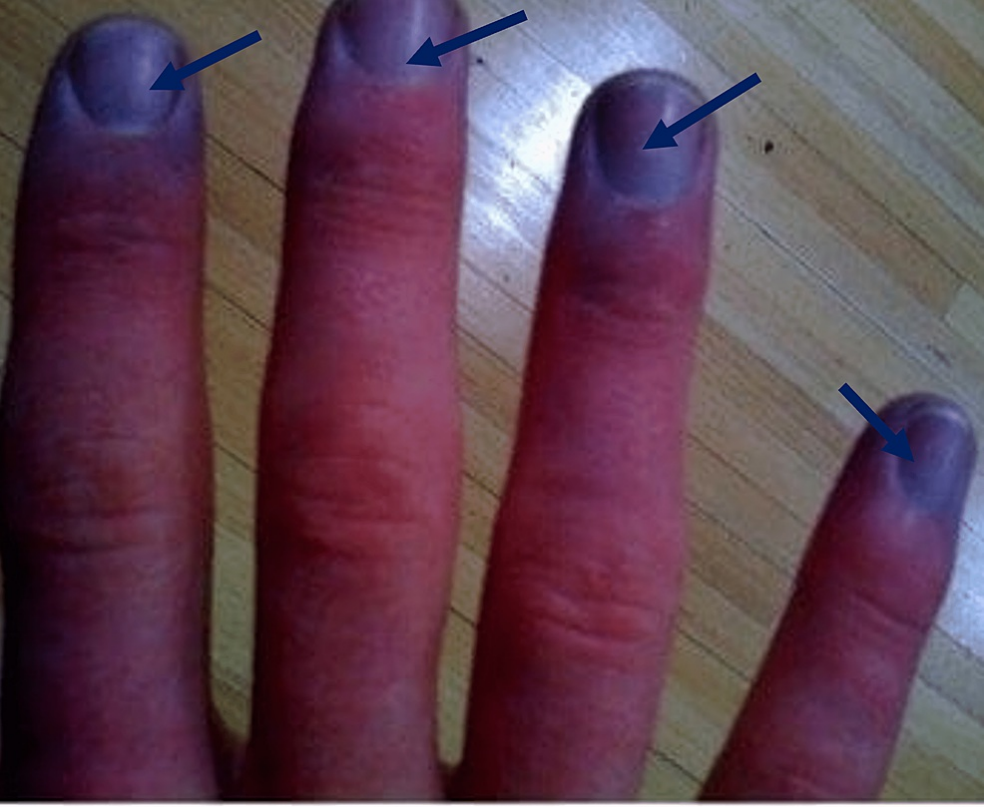
Peripheral cyanosis involving the fingertips

On examination, she did not have pallor, icterus, or pedal edema. There was central and peripheral cyanosis (Figure 1). She was afebrile, with a pulse rate of 132/minute, blood pressure of 100/70 mmHg, and respiratory rate of 32/minute with accessory muscles of respiration working. Her oxygen saturation (SaO_2_) on ambient air was 75%. Neurological examination revealed a restless, disoriented, and irritable person with a Glasgow Coma Scale (GCS) score of 12/15 (E4M5V3). Her pupils were normal in size, and reacting to light. There was no focal neurological deficit and her neck was soft. The rest of the systemic examination was normal.

As the patient was hypoxic, she was given supplemental oxygen initially by high-flow nasal oxygen (HFNO) at 20 liters/minute, which was later increased to 40 liters/minute. However, her SaO_2_ showed only a marginal improvement, to 84%. Her arterial blood gas (ABG) sample taken for analysis was dark brown. It revealed a pH of 7.39, partial pressure of carbon dioxide (PCO_2_) of 43.8 mmHg, partial pressure of oxygen (PaO_2_) of 427 mmHg, SaO_2_ of 98%, lactate of 1.6 mmol/L, fraction of methemoglobin (FMetHb) of 45.6%, and bicarbonate (HCO_3_) of 26.2 mmol/L. Given the discrepancy between SaO_2_ by pulse oximeter and normal PaO_2_ and SaO_2_ as well as high methemoglobin in ABG, and in the context of the consumption of 15 tablets of dapsone, a diagnosis of methemoglobinemia was made.

Hospital course

The patient was subsequently intubated and mechanically ventilated with pressure-regulated volume control (PRVC) mode and a fraction of inspired oxygen (FiO_2_) of 1%. She was treated with 0.1% methylene blue intravenously (IV) at a dose of 1 mg/kg body weight (total 50 mg) diluted in 100 ml of normal saline over five minutes in addition to other supportive measures. Her FMetHb after one hour was 35.3%. Hence, she was given a second dose of methylene blue 50 mg IV. The patient's basic investigations on hospital admission are presented in Table 1.

**Table 1:** Blood investigations on hospital admission TLC: total leukocyte count; LDH: lactate dehydrogenase; ALT: alanine transaminase; AST: aspartate aminotransferase; ALP: alkaline phosphatase; PT: prothrombin time; CPK: creatine phosphokinase; TSH: thyroid-stimulating hormone

Parameters	On admission	Normal range
Hemoglobin (gm/dL)	8.5	11.5–16.5
TLC (cells per mm^3^)	9,900	4,000–11,000
Neutrophils (%)	72	60–70
Lymphocytes (%)	14	20–30
Platelet count (cells per mm^3^)	150,000	150,000–450,000
Serum LDH (U/L)	433.7	140–280
Total serum protein (gm/dL)	6.69	6.6–8.3
Serum albumin (gm/dL)	3.97	3.5–5.2
Serum globulin (gm/dL)	2.72	2.5–3.5
Serum creatinine (mg/dL)	0.9	0.5–1.5
Total bilirubin (mg/dL)	1.68	0.2–1
Indirect bilirubin (mg/dL)	1.12	0.1–0.5
ALT (U/l)	18.2 U/L	5–40
AST (U/l)	40.3 U/L	5–45
ALP (U/l)	77.8 U/L	35–125
PT (seconds)	11.2	11–16
CPK (U/L)	202	40–308
Serum sodium (mmol/L)	148	135–145
Serum potassium (mmol/L)	4.2	3.5–5
Serum iron (mcg/dL)	68	60–170
Serum ferritin (mcg/L)	242	15–300
Serum folate (ng/ml)	14.6	2.7–17
Serum B_12_ (pg/ml)	478	160–950
Serum TSH (mIU/L)	1.87	6.5

There was evidence of hemolytic anemia (dapsone-induced). Her glucose-6-phosphate dehydrogenase (G6PD) level was normal and the direct antiglobulin test (DAT) and indirect Coomb’s test were negative. Her serial ABGs were as shown in Table 2.

**Table 2:** Serial ABGs from admission to discharge pH: potential of hydrogen; PCO_2_: partial pressure of carbon dioxide; PO_2_: partial pressure of oxygen; SaO_2_: oxygen saturation; FMetHb: fraction of methemoglobin; HCO_3_: bicarbonate

Arterial blood gas analysis	Day 1 (FiO_2_ 0.6%)	Day 2	Day 4	Day 6	Day 8
pH	7.44	7.37	7.35	7.37	7.42
PCO_2_ (mmHg)	39.9	45.9	53.7	45.9	36.3
PO_2 _(mmHg)	265	361	233	261	135
SaO_2 _(%)	95.0	97.0	96.3	97.0	97.3
FMetHb (%)	45.4	25.3	21.4	16.3	10.2
HCO_3 _(mmol/L)	27.0	26.2	28.9	26.2	23.3

**Figure 2:**
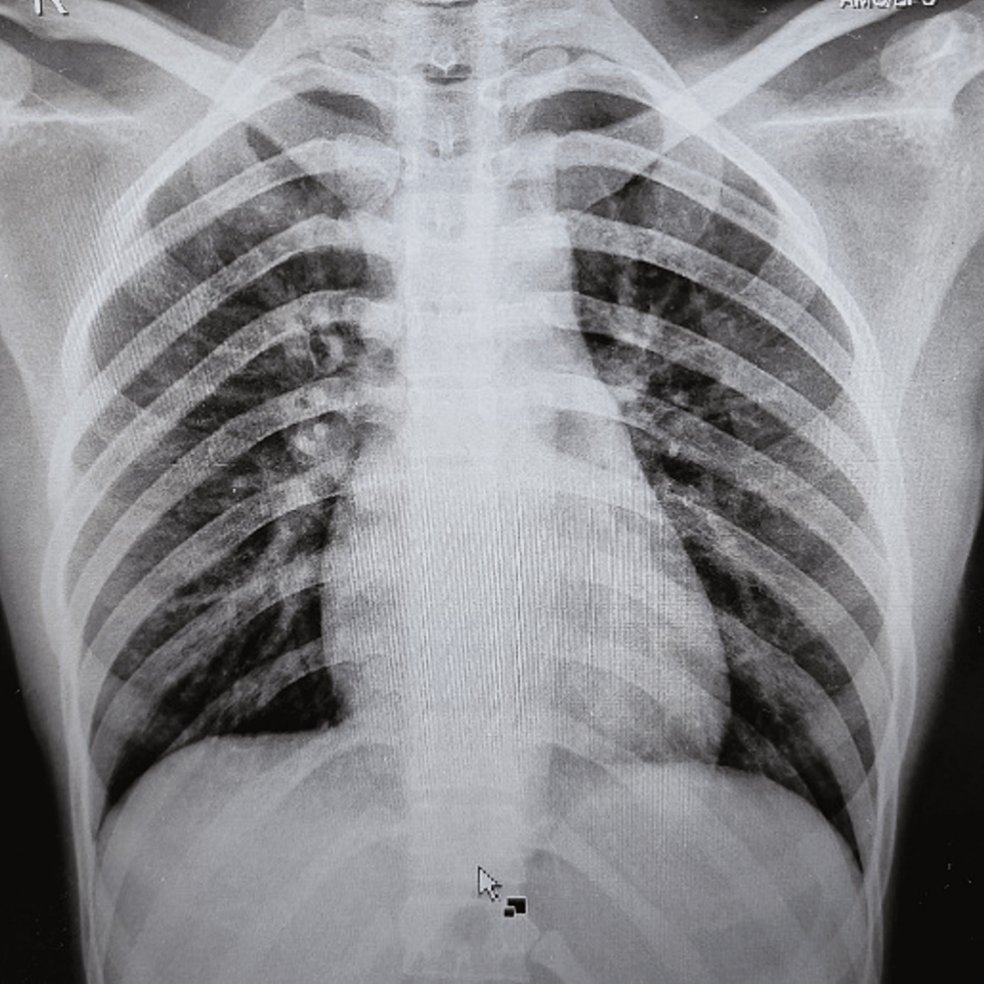
Chest radiograph - PA view showing normal lung fields except for a few calcified lymph nodes PA: posteroanterior

 Her chest radiograph (Figure 2) and echocardiography were normal.

An electrocardiogram (ECG) showed sinus tachycardia. A final diagnosis of dapsone-induced hemolytic anemia with methemoglobinemia was made. Over the next seven days, her FMetHb gradually decreased to 10.6%. She received a total of 250 mg of methylene blue. She was subsequently discharged against medical advice (with endotracheal tube in situ) on the eighth day and was taken to a different center for further management. There she was ventilated and received methylene blue for two more days. When her FMetHb decreased to less than 2%, she was extubated and subsequently discharged.

## Discussion

Methemoglobinemia is caused by abnormal hemoglobin characterized by the ferric form of iron (Fe^3+^) in the heme moiety, resulting in a conformational change in hemoglobin, which leads to impaired oxygen-carrying capacity and the release of oxygen to the tissues [1]. Oxidation of iron also causes a shift of the oxygen-hemoglobin dissociation curve to the left, functional anemia, and tissue hypoxia. Normal red cells maintain MetHb levels of less than 1% by two enzymatic pathways namely cytochrome-b5 reductase and nicotinamide adenine dinucleotide phosphate hydrogen methemoglobin (NADPH-MetHb) reductase, which predominate during oxidative stress [2]. Acquired causes of methemoglobinemia include exposure to toxic amounts of oxidizing agents like benzocaine, lignocaine, dapsone, sulphonamides, chloroquine, and aniline dyes [3].

The clinical features vary from being asymptomatic to a potentially life-threatening condition, depending upon the level of methemoglobin, rate of its accumulation, and baseline hemoglobin of the patient. Cyanosis, which was present in our case, develops at a serum methemoglobin level of 15%. Levels of 30-45% result in headache, light-headedness, fatigue, weakness, and palpitation while levels above 60% result in cardiac arrhythmias, breathlessness, seizures, and coma. Methemoglobin levels greater than 70% are fatal [4,5]. The diagnosis is based on the history, clinical symptoms, and elevated blood methemoglobin level with an ABG machine that has the facility for co-oximetry.

In our case, the diagnosis was quite evident based on the patient's history of consumption of 15 tablets of dapsone and her high blood MetHb level. Since all ABG machines do not have this provision, clinicians should be aware of the "saturation gap" - the difference in oxygen saturation (SaO_2_) given by the ABG machine and that by pulse oximeter [5]. While the pulse oximeter underestimates the oxygen saturation in the presence of MetHb, the ABG machine calculates it from the oxyhemoglobin dissociation curve [5]. Furthermore, the presence of central cyanosis in the absence of cardiac or respiratory cause, and a normal PaO_2_ level should make one suspect the presence of abnormal hemoglobin. The blood may appear "chocolate brown" in color (chocolate cyanosis) in the case of methemoglobinemia.

Dapsone is a sulfone antibiotic that interferes with folate metabolism. It is used in the treatment of a variety of conditions like Hansen’s disease, pneumocystis jirovecii pneumonia (PJP), toxoplasmosis, resistant immune thrombocytopenia, and dermatitis herpetiformis [6]. Metabolites of dapsone - dapsone hydroxylamine and monoacetyldapsone hydroxylamine - can cause methemoglobinemia and Heinz body hemolytic anemia from oxidant stress. It is the most commonly implicated offending agent. Doses above 200 mg/day are associated with methemoglobinemia in a dose-dependent manner. Our patient had consumed 1500 mg of dapsone. If a stomach wash had been given within one hour of consumption, it might have reduced some absorption.

The management of the condition includes the withdrawal of the offending drug and administering the specific antidote methylene blue. It is given in an asymptomatic patient when FMetHb is more than 30% and more than 20% in a symptomatic patient [6,7]. A dose of 1-2 mg/kg body weight given intravenously over five minutes leads to a rapid reduction of MetHb levels. Methaemoglobin is reduced by NADPH (reduced form of nicotinamide adenine dinucleotide phosphate)-dependent methemoglobin reductase to leukomethylene blue, which acts as an electron donor to MetHb, thereby reducing it back to hemoglobin [8]. It utilizes the normally dormant G6PD pathway. As it is a potent monoamine oxidase inhibitor, it should not be given to patients taking serotonergic agents due to the risk of serotonin syndrome [3]. If given repeatedly, it may cause rebound methemoglobinemia. Vitamin C in high doses (10 grams, six-hourly IV) is given to G6PD-deficient patients as methylene blue may be ineffective and cause hemolysis [8]. Other treatment options include hyperbaric oxygen and red cell exchange transfusion in refractory cases.

In a case reported by Moulis et al., MetHb levels fell from 17 to 4% in 20 minutes after 2 mg/kg IV administration of methylene blue. However, after 10 days, the patient had rebound methemoglobinemia at 14% [9]. In our case, the fall in MetHb concentration was slow, probably due to the larger amount of absorbed drug and slow elimination due to the long half-life of dapsone. Most of the cases published earlier in the literature were caused by the cumulative effect of dapsone consumed for therapeutic purposes [3,5,7,8] and they all responded to IV methylene blue. In our case, the total dose of methylene blue required was 350 mg for the methemoglobin level to normalize. In an earlier case report by Shadnia et al, the patient had consumed 2 grams of dapsone with suicidal intent, and the MetHb level was 38%. She also developed hemolytic anemia requiring blood transfusion and acute hepatitis. She ultimately responded to 50 mg of methylene blue IV and 12-hourly vitamin C 1000 mg IV [10]. The drug history, however, was not evident on admission.

## Conclusions

Methemoglobinemia is a potentially life-threatening condition. If not detected early, it can result in death. It initially presents with non-specific symptoms. With dapsone being increasingly used for therapeutic purposes, this side-effect of dapsone has to be considered in any patient presenting with unexplained symptoms like confusion, agitation, palpitation, etc. With higher levels of MetHb, the saturation gap in oxygen saturation between ABG and pulse oximetry should raise suspicion, which can be confirmed by the level of methemoglobin shown by an ABG machine that has the facility for co-oximetry. Methylene blue is the drug of choice for reducing the level of methemoglobin. Clinicians should be aware of this dreaded complication of dapsone, as early recognition and timely intervention can result in favorable outcomes.
